# Notes and Notices

**Published:** 1882-06

**Authors:** 


### NOTES AND NOTICES.

REMOVALS.—Dr. E. P. Hussey has removed to Buffalo, N. Y. Address, 55, The Circle. Dr. Constantine Lippe has changed his residence and offices from 110 W. 40th Street to 68 W. 50th Street (N. Y.), where he has purchased a handsome property. Dr. J. E. Cross has returned from Florida to West Eaton, N. Y. Dr. George H. Clark, at the request of friends, has removed to Germantown, to fill the place left vacant by the decease of Dr. Thomas Moore. The residents of Germantown are to be congratulated.

LONG BRANCH.—Dr. Thomas Wildes, of New York, will act as resident physician during the coming summer season, at the Howland House, Long Branch.

WORSE YET.—Prof. T. F. Allen laments that so few homœopathic physicians can correctly pronounce the names of their drugs. Many patients have a much more serious charge; they lament that so few can *prescribe* their drugs correctly!

THE KIND VIRGINIA WANTS.—"We want no more men who prescribe tonics, purgatives and blisters to represent Homœopathy in Virginia, but men who are fearless enough to prescribe according to the law of the similars, and who, at least, have *read* the Organon."—M. E. Douglass, in the *Weekly Counselor*.

A FARCE AND A TRAGEDY.—To understand this farce (of which we can give only a few points) it is necessary to first state the *plot*. The scene is laid in a homœopathic college, actors are said to be professors of Homœopathy, audience are students, who have paid said professors to teach them *Homœopathy*.

*First Professor*—I cure my cases of intermittent fever by rigidly adhering the "principle" S. S. C. in all cases. I individualize each case closely and find quinine to be the remedy. (Audience take notes for future use.)

*Second Professor*—I have a weakness for quinine. If I could get along without quinine, as some claim to do, I could get along without Homœopathy altogether.

*Third Professor*—If I had to depend, in the treatment of intermittent fever, on either the remedy or hygiene alone, I most certainly would prefer to depend on the hygiene.

*First Professor*—I only give quinine or china in chills when it is homœopathically indicated, which is pretty nearly always. "I wish to tender my congratulations to the society on the very remarkable degree of unanimity exhibited in this discussion!"

The tragedy of the play is furnished by the students carrying out in practice the precepts of their professors.

